# Unexpected Synthesis of a Bulky Bis-Pocket A_3_B-Type *Meso*-Cyano Porphyrin

**DOI:** 10.3390/molecules22111941

**Published:** 2017-11-09

**Authors:** Ze-Yu Liu, Mian HR Mahmood, Jian-Zhong Wu, Shu-Bao Yang, Hai-Yang Liu

**Affiliations:** 1 School of Chemistry and Environment, South China Normal University, Guangzhou 510006, China; liuzeyu00@163.com; 2 Department of Chemistry, South China University of Technology, Guangzhou 510641, China; mianhrm01@ue.edu.pk (M.H.M.); yangshubao2008@sina.com (S.-B.Y.); 3 Department of Chemistry, University of Education, Lahore 54770, Pakistan

**Keywords:** *meso*-cyano porphyrin, One-pot synthesis, pyrrole-aldehyde condensation, trifluoroacetic acid

## Abstract

A one-pot synthesis of bulky bis-pocket A_3_B-type *meso*-cyano porphyrin, 5-cyano-10,15,20-tris(2,4,6-triphenylphenyl)porphyrin, has been accomplished via trifluoroacetic acid (TFA) catalyzed condensation of pyrrole and 2,4,6-triphenylbenzaldehyde in an acceptable yield of about 4%. DDQ served as oxidant and the cyanating agent.

## 1. Introduction

The diversity in the synthetic chemistry of porphyrinoid compounds derived from the conventional pyrrole-aldehyde condensation continues to fascinate the scientific community [1,2]. The versatile self-assembly process, generating a ring-expanded or -contracted porphyrinoid, generally ends up with or without oxidative handling depending on the solvating media [3]. This is well illustrated from the recent reports in the facile synthesis of *meso*-patterned corroles [4,5,6,7] and porphyrins [8,9], expanded and isomeric porphyrins [1,2,10], and related porphyrin-type macrocyclic compounds [11].

**Figure 1:**
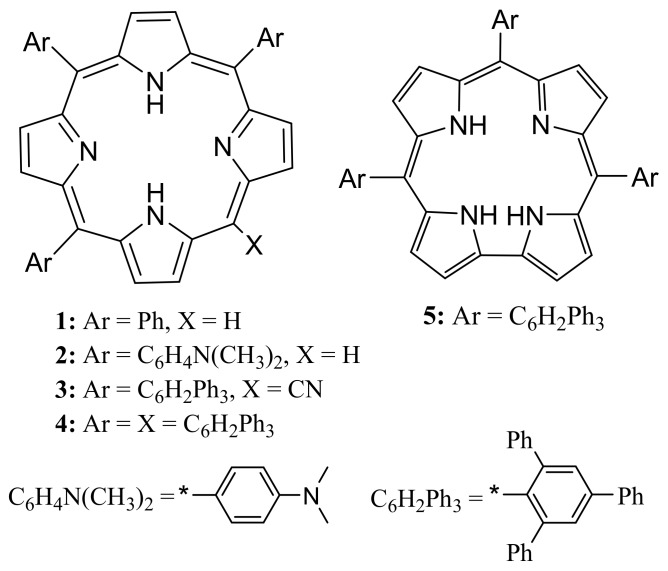
Molecular structures of porphyrins and corrole.

Despite this fact, the one-pot synthesis of A_3_-type porphyrin such as **1** (Figure 1) from a single aldehyde precursor is still a relatively less explored aspect. This may be attributed to the extreme reactivity of pyrrole and aldehyde units toward each other, which usually resulted in the formation of their comparatively more stable symmetrical counterparts [12]. This is indeed responsible for broadening the scope and general ease in the synthesis of *meso*-tetraaryl porphyrins in recent years [13].

The characteristic structure here refers to the presence of aryl groups at three of the four available *meso*-positions, while the fourth *meso*-position may serve as a methine-bridge only, as in A_3_-type porphyrin, or may undergo subsequent functionalization to give A_3_B-type porphyrin. Such porphyrins have been extensively employed as synthons for various porphyrin building blocks and as surrogates for the naturally occurring tetrapyrrolic macrocycles [14,15]. The literature reports have emphasized the synthesis of A_3_-type porphyrins via *meso*-unsubstituted dipyrromethane procedure [14,15,16,17], which is often accomplished in multi-step process and with low yields. A noteworthy observation in this regard is the interconversion of *trans*-A_2_B corrole into the corresponding A_3_-type porphyrin; simply via prolonged stirring of the methanol or benzene solution of the corrole [18]. A 2π + 2π cycloaddition reaction with subsequent formation of a spirocyclobutane was proposed to be the intermediate in such a conversion. We recently reported the first example of the direct synthesis of A_3_-type porphyrin (**2**, Figure 1) via one-pot condensation of pyrrole and highly electron-rich aldehyde, 4-(*N*,*N*-dimethylamino)benzaldehyde [19], in the presence of trifluoroacetic acid (TFA). Here, we want to report the unprecedented one-pot synthesis of bulky bis-pocket A_3_B-type *meso*-cyano porphyrin (**3**, Figure 1). The supported spectroscopic characterization and the plausible reaction mechanism are also presented.

## 2. Results and Discussion

Our interest in the synthesis of porphyrinoids originated from peculiar electronically and sterically crowded aldehydes was due to their intrinsic ability to serve as excellent mechanistic probe for the oxygenation reactions [20,21,22]. Porphyrins obtained from *ortho*-disubstituted aldehydes are typically employed as catalyst in order to avoid the formation of µ-oxo and µ-peroxo dimer intermediates during the catalytic reaction [23]. This approach was exploited in the synthesis of sterically crowded bis-pocketed porphyrin, 5,10,15,20-tetrakis(2,4,6-triphenylphenyl)porphyrin **4** (H_2_TTPPP) [24], and corrole, 5,10,15-Tris(2,4,6-triphenylphenyl)corrole **5** (H_3_TTPPC) [25] that have non-polar pockets on both faces of the macrocycle.

**Figure 2:**
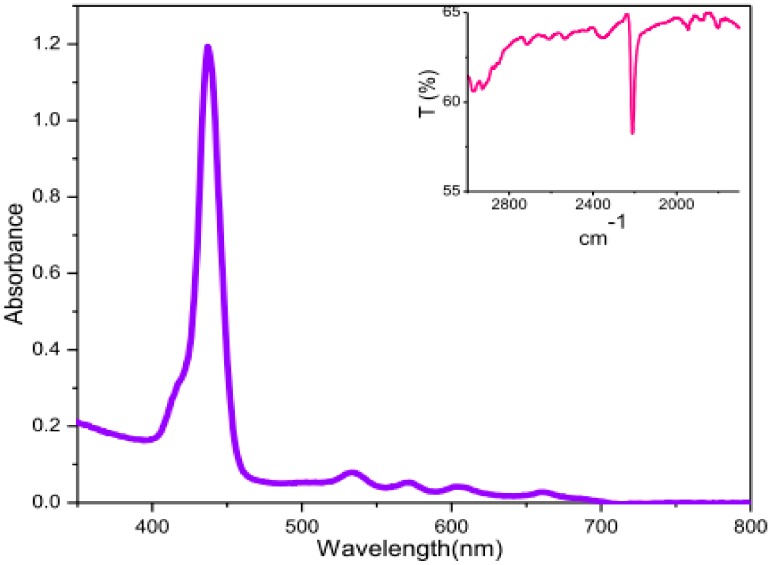
UV-Vis spectrum of 5-cyano-10,15,20-tris(2,4,6-triphenylphenyl)porphyrin 3 in dichloromethane. The inset is the partial IR spectrum showing the characteristic –C≡N stretching absorption at 2211 cm^−1^.

Suslick et al. reported the first synthesis of porphyrin **4** (H_2_TTPPP) in 1% yield via slow addition of a stochiometric amount of pyrrole diluted in xylenes to a refluxing propionic acid solution of the corresponding aldehyde [24] or by using 2,4,6-collidine as solvent at elevated temperature [26]. The inspirations for our attempts to improve the yield of the bulky bis-pocket porphyrin came from the independent work of Lindsey [8,9] and Drenth [27]. They observed that condensation of pyrrole and arylaldehyde under mild conditions in inert atmosphere using higher dilutions in chlorinated solvents (CH_2_Cl_2_ or CHCl_3_) and strong acid as catalysts significantly improved the yield of porphyrins with bulky substituents at the *ortho* positions. We have previously observed that TFA catalyst may dramatically improve the yield of corrole **5** (H_3_TTPPC) from the condensation of 2,4,6-triphenylbenzaldehyde and its dipyrromethane [25]. However, the application of TFA in condensation of the same aldehyde and pyrrole in dichloromethane solvent did not deliver any observable yield of the symmetrical porphyrin **4** (H_2_TTPPP). We then turned our attention to the use of boron trifluoride etherate (BF_3_·Et_2_O) as a catalyst introduced by Lindsey in 1986 [8], which proved to be the most effective acid catalyst for the synthesis of porphyrinoids in the recent years. Recently, we also found BF_3_⋅Et_2_O was efficient in the synthesis of bulky multibrominated corrole [28]. A previous report described the unsuccessful attempts for the synthesis of porphyrin **4** (H_2_TTPPP) using BF_3_·Et_2_O catalyst [27], probably due to the excessive dilution and longer reaction time. We also failed to get porphyrin **4** (H_2_TTPPP) by using the same catalyst and observed TFA was more active than BF_3_·Et_2_O in the current reaction as indicated by the color change of the mixture. A more careful investigation of TFA catalyzed condensation of 2,4,6-triphenylbenzaldehyde and pyrrole directed us to the current unexpected finding of one-pot synthesis of A_3_B-type *meso*-cyano porphyrin **3** (H_2_TTPPPCN). We observed that stirring the dichloromethane solution (10^−3^ M) of equimolar 2,4,6-triphenylbenzaldehyde and pyrrole in the presence of TFA catalyst, followed by quenching with triethylamine and oxidation with DDQ, and the chromatographic work-up mainly provided a dark green colored product with an isolated yield of about 5%. It turned out to be 5,10,15-tris(2,4,6-triphenylphenyl)corrole **5** (H_3_TTPPC) [25]. At the same time, we had isolated a trace amount of porphyrin that showed a sharp Soret band at 437 nm and four Q-bands in the region of 530–670 nm (Figure 2).

**Figure 3:**
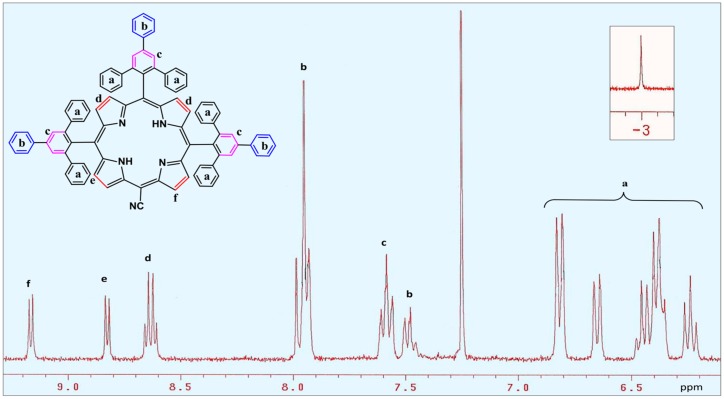
Partial ^1^H-NMR spectrum of porphyrin **3** showing the aromatic region, inset shows the inner N-H protons.

Surprisingly, the appearance of mass ion peak at 1248.3 (Figure S1) in the FAB-MS spectrum corresponds to neither porphyrin **4** (H_2_TTPPP) nor corrole **5** (H_3_TTPPC). The difference of more than 300 mass units between the observed mass and the expected symmetrical porphyrin **4** suggested the absence of one *meso*-triphenylphenyl group in the compound. Nonetheless, even on considering the observed compound as A_3_-type porphyrin, its mass was 26 mass units more than the expected mass. The ^1^H-NMR spectrum pattern of the compound (Figure 3) was in accordance with the typical spectrum of an A_3_B-type porphyrin [19], showing a peak at −3.00 ppm corresponding to two inner N-H protons. However, the absence of C-H peak at approximately 10 ppm in the NMR spectrum (Figure S2) was indicative of the fact the fourth *meso*-carbon was not free [18] but attached with a substituent other than that originating from the starting aldehyde. In the absence of any external source in our reaction system serving as an additional *meso*-substituent, this observation was truly unexpected. 

We speculated that another group that could serve as the fourth *meso*-substituent in the reaction system in addition to aldehyde, with a mass equivalent to 26 units, was the ‒CN group from the DDQ. This hypothesis was supported by IR spectrum that indicated a sharp ‒C≡N stretching absorption at 2211 cm^−1^ (Figure 2, inset). The confirmation of the above ambiguity also came from the HR-MS analysis (Figure S4), which provided a more strong evidence of the product A_3_B-type *meso*-cyano porphyrin **3** (H_2_TTPPPCN), where the three *meso*-substituents came from the aldehyde, and the fourth cyano group might come from the oxidant DDQ [29]. 

**Figure 4:**
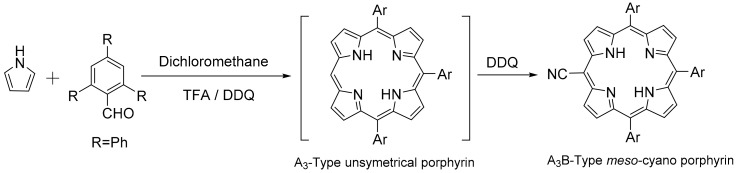
Plausible reaction route for the one-pot synthesis of A_3_B-type *meso*-cyano porphyrin **3** (H_2_TTPPPCN).

The serendipitous one-pot synthesis of bulky bis-pocket A_3_B-type porphyrin in the case of 2,4,6-triphenylbenzaldehyde implies that more sterically crowded aldehydes may undergo similar condensation process to that of the highly electron-rich aldehyde [19], as reported recently. Although the mechanism is still uncertain, a feasible pathway seems to be the formation of a tetrapyrrolic precursor, where the steric hinderance provided by the bulky groups does not facilitate the attachment of the fourth *meso*-substituent. It is noteworthy that the same steric factors might be responsible for the unexpectedly low yield (1%) of the corresponding symmetrical porphyrin analogue [24]. The resulting corrole intermediate may undergo 2π + 2π cycloaddition reaction, followed by the formation of a spirocyclobutane, which on air-oxidation may generate an A_3_-type porphyrin devoid of one *meso*-substituent [18]. The addition of DDQ at this stage completes the oxidation process and causes the substitution of the hydrogen with cyano group at the sole available *meso*-position of the porphyrin, resulting in the formation of A_3_B-type *meso*-cyano porphyrin **3** (H_2_TTPPPCN) (Figure 4).

While *meso*-cyano porphyrins are generally obtained via peripheral functionalization of the preformed porphyrins [30,31], a convenient one-pot approach where the oxidant itself serves as an efficient cyanating agent has been reported [29]. It is well documented in the literature that DDQ can interact with the free base porphyrins through inner N-H protons and coordinates exclusively from above and below the plane of the porphyrin, forming 2:1 molecular complexes between DDQ and porphyrin, respectively [32]. For the current observation, it seems more likely that an adduct is first formed between DDQ and porphyrin, which then transfers the cyanide ion [29] from DDQ at the *meso*- position of the porphyrin because of its higher susceptibility to attack, thus giving a *meso*-cyano-substituted porphyrin. The cyano-substituted porphyrins are considered to be among the most useful precursors for subsequent transformations because the nitrile group is quite amenable to other functionalities such as aldehydes, amines, amides, and acid derivatives [33]. Thus, the one-pot synthesis of A_3_B-type cyano-substituted porphyrin may have practical usage in the preparation of functional porphyrins. After optimization of the reaction conditions, the yield of **3** (H_2_TTPPPCN) may reach about 4%. This yield is acceptable, considering the low yield of ~1% of H_2_TTPPP (**4**) [24]. For a typical procedure for **3**, see experimental section.

## 3. Experimental Section

The formation of bulky A_3_B-type *meso*-cyano porphyrin followed the general procedure for porphyrin synthesis developed by Lindsey et al. [9]. Typically, equimolar amounts of 2,4,6-triphenylbenzaldehyde (250 mg, 0.75 mmol) and pyrrole (52 µL, 0.75 mmol) were mixed together in 100 mL of dry dichloromethane in a 250 mL round bottom flask. To this mixture an aliquot of trifluoroacetic acid (TFA, 150 µL) was added, and the solution was stirred overnight. After that, 200 mg of 2,3-dichloro-5,6-dicyano-1,4-benzoquinone (DDQ) were added to the reaction mixture and stirred for 1 h. Then, 300 µL of trimethylamine was added to neutralize the solution and the mixture was stirred for another 1 h. The reaction was monitored by thin-layer chromatography and UV-Vis spectroscopy. After the completion of reaction, the solvent was dried in vacuum, and the resulting crude product was purified by chromatography on silica gel column using dichloromethane/hexane (1/2) as eluent and recrystallized from methanol /dichloromethane (1/1, *v*/*v*) to afford 12 mg of **3** (H_2_TTPPPCN) (Yield, 3.8%). UV-Vis (CH_2_Cl_2_): *λ*_max_/nm 438, 534, 571, 606, 662; ^1^H-NMR (300 MHz, CDCl_3_): *δ* 9.16 (d, *J* = 4.6 Hz, 2H), 8.81 (d, *J* = 4.6 Hz, 2H), 8.63 (AB system, *J* = 4.8 Hz, 4H), 7.96 (m, 12H), 7.57 (apparent t, *J* = 7.8 Hz, 6H), 7.48 (m, 3H), 6.8 (d, *J* = 6.6 Hz, 8H), 6.64 (d, *J* = 7.5 Hz, 4H), 6.49−6.34 (m, 14H), 6.23 (t, *J* = 7.8 Hz, 4H), −3.00 (s, 2H); FAB-MS: *m/z* 1248.3; HR-MS calcd exact mass (C_93_H_62_N_5_), 1248.5005; found, 1248.5007 [M + H]^+^.

## 4. Conclusions

In summary, we present the first direct synthesis of very sterically crowded bis-pocket A_3_B-type *meso*-cyano porphyrin from a single aldehyde precursor in a one-pot pyrrole-aldehyde condensation. It is clear that the electronic and steric features of aldehydes, as well as changing acid catalyst and reaction conditions, such as dilution, affect the nature of the final porphyrinoid product. It may also demonstrate that macrocyclic species may undergo reorganization within the reaction system to attain relatively stable configuration on grounds of electronic and steric factors. Further studies based on exploring the scope of various aldehydes within the current synthetic methodology, along with their optimization, are currently in progress in our laboratory.

## Supplementary Materials

Experimental data for the bulky A_3_B-type *meso*-cyano porphyrin are available online.
