# Supplementary material for: Unexpected Synthesis of a Bulky Bis-Pocket A3B-Type Meso-Cyano Porphyrin
Source: Molecules. 2017 Nov 9;22(11):1941. doi: 10.3390/molecules22111941 (PMC6150394; doi:10.3390/molecules22111941)
Supplement: Supplementary file 1 [file molecules-22-01941-s001.pdf]

Supporting Information for

Unexpected synthesis of a bulky bis-pocket A_3B-type *meso*-cyano porphyrin

Ze-Yu Liu[1], Mian HR Mahmood[2,3], Jian-Zhong Wu[1,*], Shu-Bao Yang[2], Hai-Yang Liu[2,*]

[1] School of Chemistry and Environment, South China Normal University, Guangzhou 510006, P.R. China.
[2] Department of Chemistry, South China University of Technology, Guangzhou 510641, P.R. China.
[3] Department of Chemistry, University of Education, Lahore 54770, Pakistan.

CONTENTS

Entry	Detail	Page no.
1	FAB-MS spectrum of porphyrin **3**	S2
2	^{1}H NMR spectrum of porphyrin **3**	S3
3	Infra-red spectrum of porphyrin **3**	S4
4	HR-MS of porphyrin **3**	S5

Corresponding Authors: Tel. & *Fax:* +86-020-39310187, E-mail: wujzh@scnu.edu.cn (J-Z. Wu); *+86-020-22236805; E-mail:* chhyliu@scut.edu.cn (H.-Y. Liu)

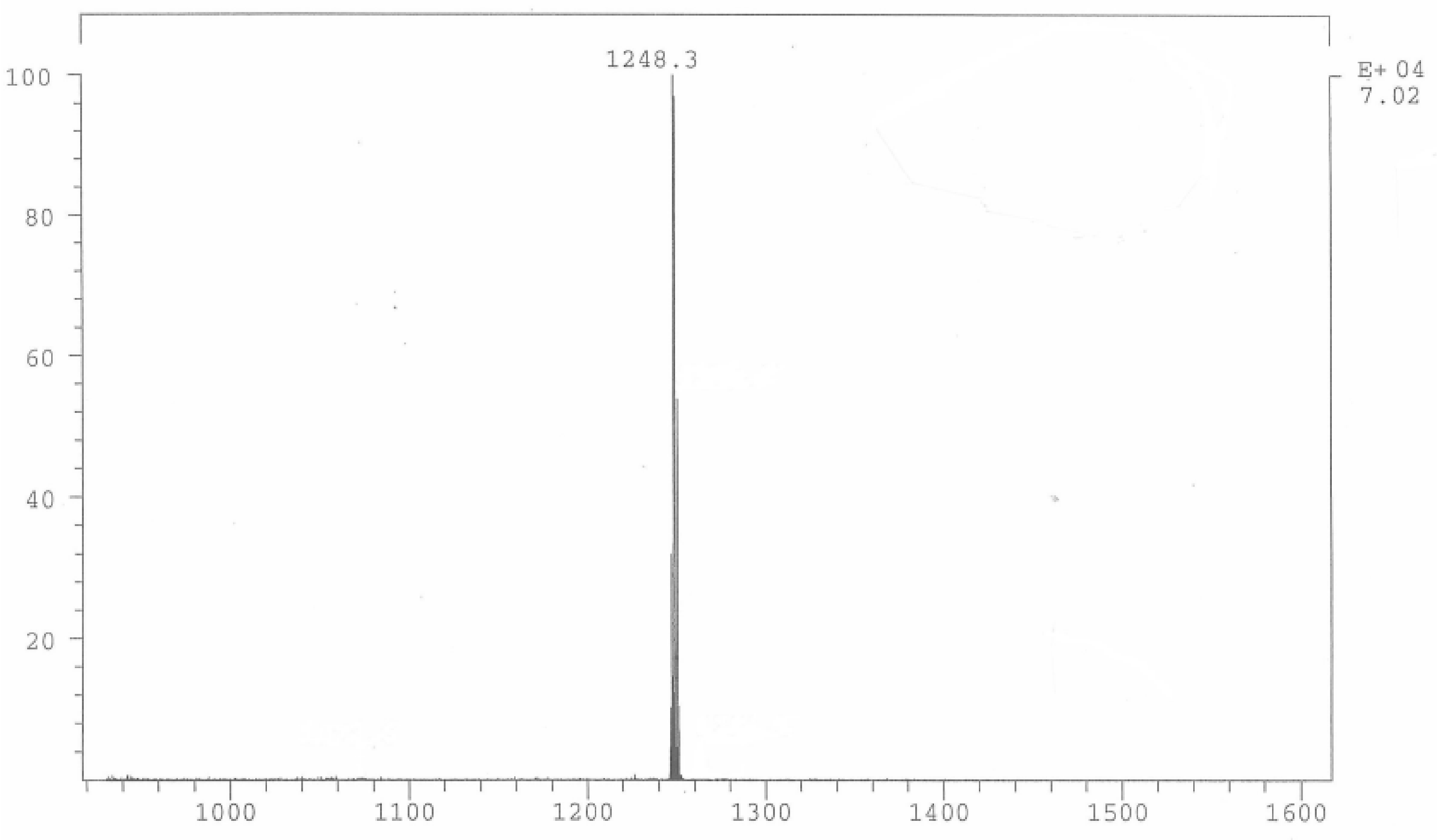

Figure S1. FAB-MS spectrum of porphyrin **3**

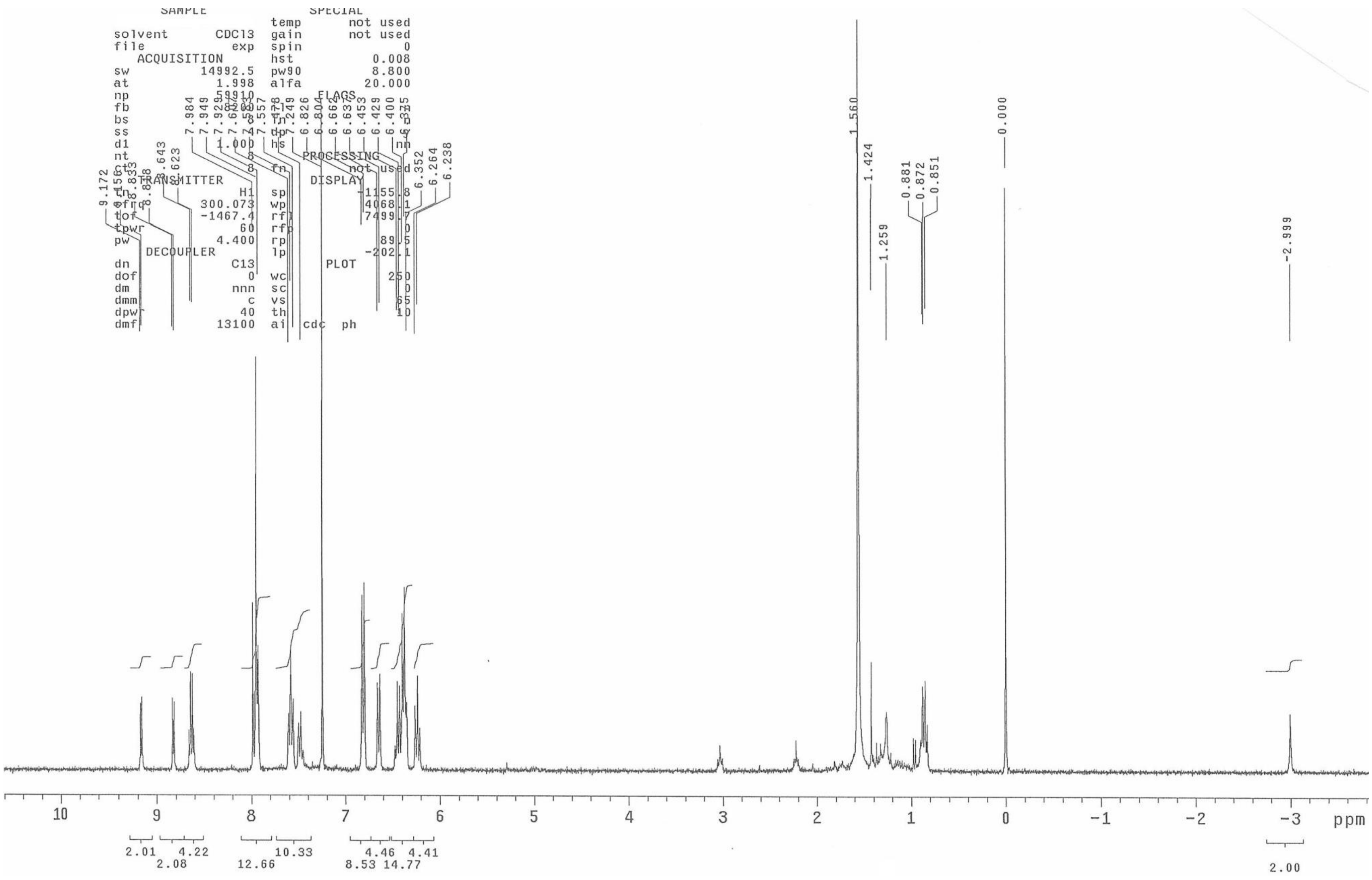

Figure S2. ^{1}H NMR spectrum of porphyrin **3**

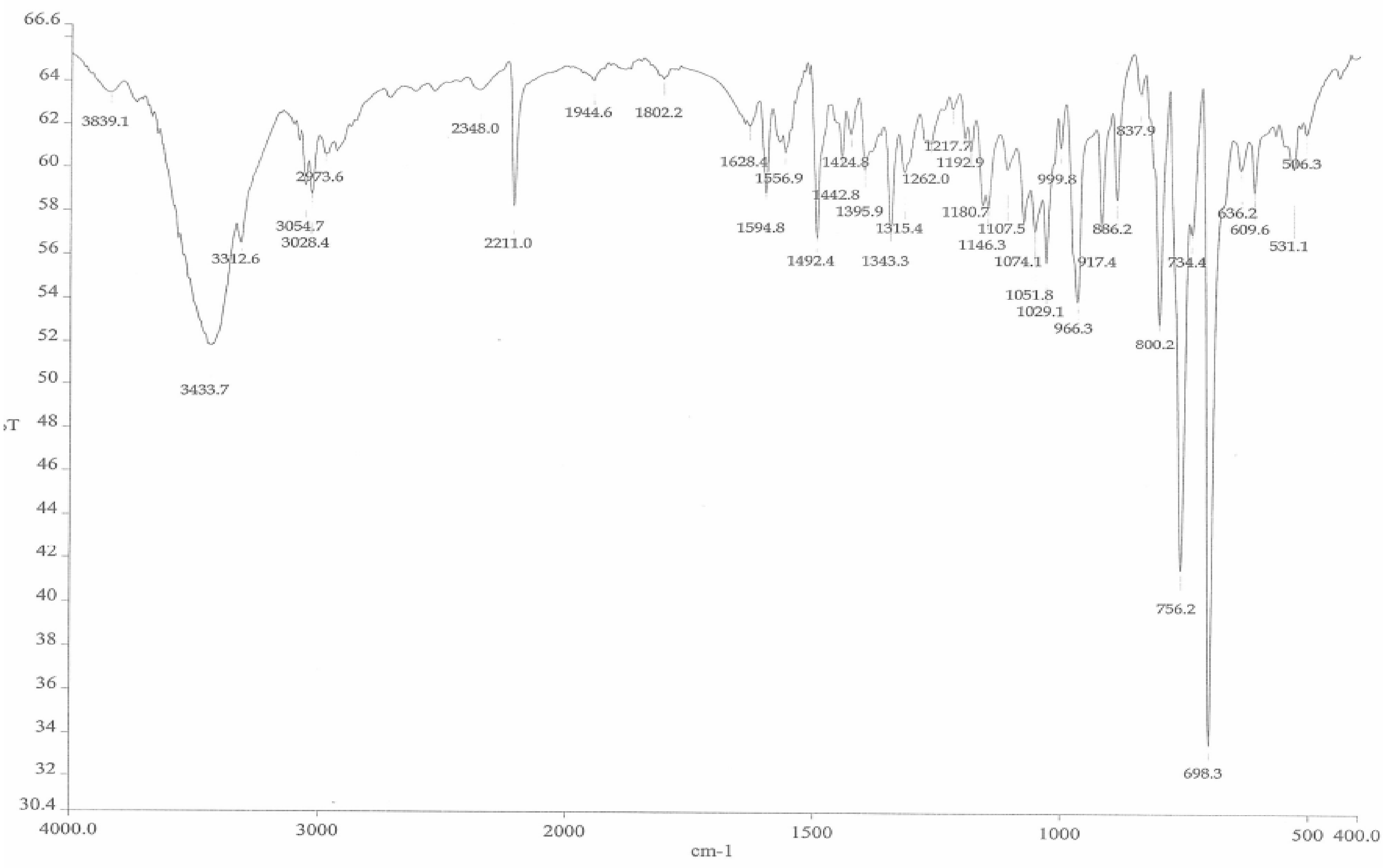

Figure S3. Infrared spectrum of porphyrin **3**

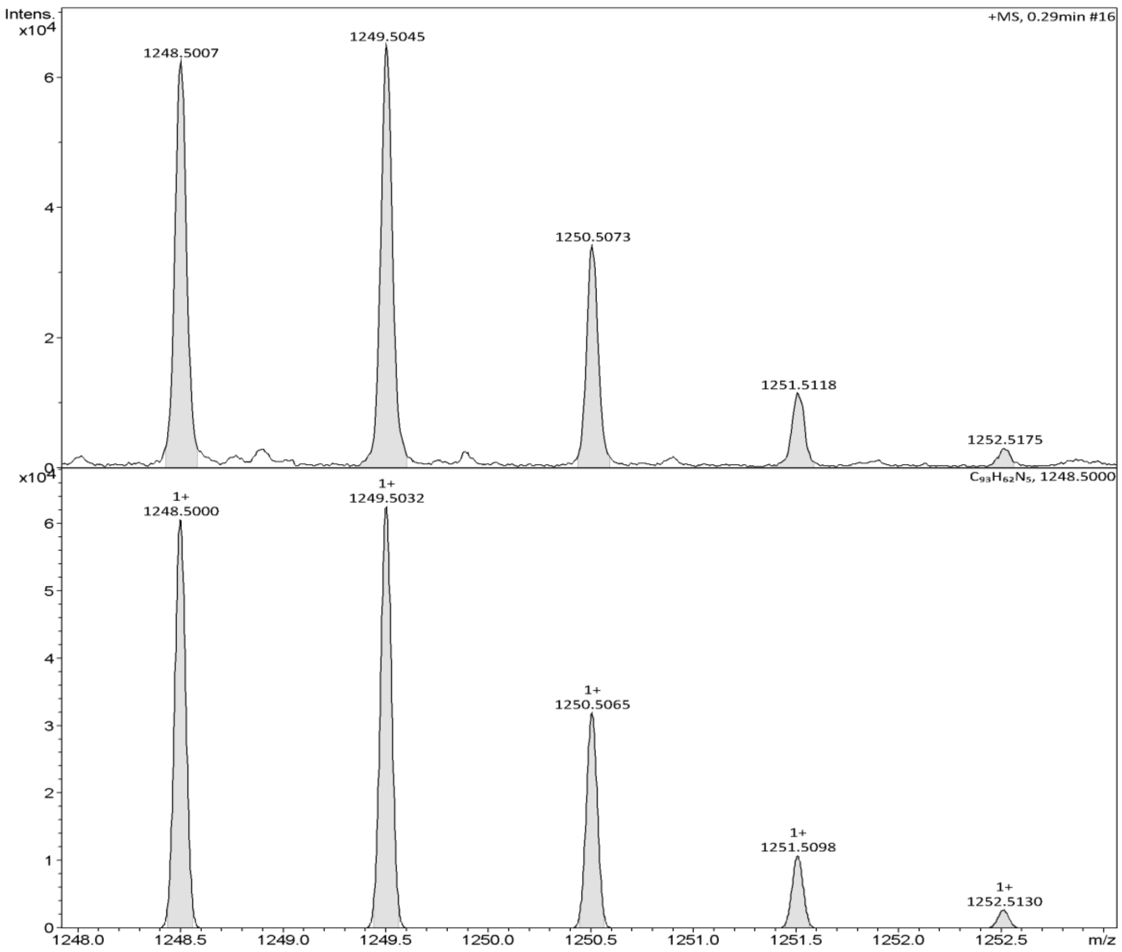

Figure S4. HR-MS of 5-Cyano-10,15,20-tris(2,4,6-triphenylphenyl)-porphyrin **3** ($H_2TTPPPCN$).
